# Long-Term Exposure to Greenspace and Cognitive Function during the Lifespan: A Systematic Review

**DOI:** 10.3390/ijerph191811700

**Published:** 2022-09-16

**Authors:** Elisabetta Ricciardi, Giuseppina Spano, Antonella Lopez, Luigi Tinella, Carmine Clemente, Giuseppe Elia, Payam Dadvand, Giovanni Sanesi, Andrea Bosco, Alessandro Oronzo Caffò

**Affiliations:** 1 Department of Educational Sciences, Psychology, Communication, University of Studies of Bari, 70122 Bari, Italy; 2 Department of Agricultural and Environmental Sciences, University of Studies of Bari, 70126 Bari, Italy; 3 Faculty of Law, Giustino Fortunato University, 82100 Benevento, Italy; 4 Barcelona Institute for Global Health, 08003 Barcelona, Spain; 5 Universitat Pompeu Fabra (UPF), 08003 Barcelona, Spain; 6 CIBER Epidemiologíay Salud Publica (CIBERESP), 28029 Madrid, Spain

**Keywords:** greenspace, cognitive functions, memory, attention, executive functions, visuospatial, Bayesian average

## Abstract

Recent advances in environmental psychology highlighted the beneficial role of greenspace exposure on cognition. We conducted a systematic review of the available studies on the association of long-term exposure to greenspace and cognitive functions across the lifespan. PRISMA guidelines and the PECOs method were applied to screen for eligible studies. Twenty-five studies from Scopus, PubMed, and PsycINFO met the inclusion criteria. Six studies were longitudinal and nineteen cross-sectional. Fifteen studies focused on schoolchildren, six studies on adults, and four on the elderly. Twenty studies used the NDVI to assess greenspace exposure and the remaining used other indexes. Eight studies employed academic achievement as the outcome, eight studies global cognition, six studies attention/executive functions, and three studies memory. The evidence was inconsistent but suggestive for a beneficial role of greenspace exposure on cognitive functions. Further studies are required, especially among adults and older people, by adopting longitudinal designs.

## 1. Introduction

Approximately 55% of the population lives in urban areas, and by 2050 it is predicted that this number will rapidly increase and about 85% of people in Europe will live in cities [1,2]. The growing urbanization influences greenspace fragmentation [3] and the spread of urban greenspace (UGS) as a part of green infrastructure (GI) which is increasing in the urban world [4]. The linking between presence and use of greenspace in urban contexts and human well-being has been of interest for a lot of studies in the field of environmental science [5,6]. Reviews and meta-analyses [7,8,9] have suggested the association of greenspace exposure with mental health in children and middle-aged and older adults. Davis et al. (2021) evaluated 45 studies and found evidence on the association between greenspace and emotional and behavioral well-being in children, such as reduction in anxiety, depression, and aggressive behavior. On the other hand, Gascon et al. (2015) found inadequate evidence for a relationship between greenspace and mental health among children and limited evidence among adults. Furthermore, Li et al. (2021), in their systematic review, found mixed results on the beneficial role of early nature exposure in mental health in later life. In addition, in a recent study, residential greenness was associated with fewer occurrences of problematic behavior in children [10]. Moreover, living in proximity to greenspace was found to be associated with a lower incidence of depressive symptoms in adolescents and young adults [11]. Other studies have shown an association between neighborhood greenness and a decrease in perceived stress [12], and residential greenness was positively associated with light-intensity physical activity among adults and older adults [13].

To explain the positive effect of greenspace on health and wellbeing, different biopsychosocial mechanisms could be hypothesized [14]. Specifically, air pollutant concentrations, such as traffic-related pollution exposure, are lower in green. The presence of greenspace has been reported to reduce levels of traffic noise, which in turn is associated with physical health, such as cognitive functions and the risk to develop neurodegenerative disorders [15,16]. Moreover, use of greenspace has also been reported to encourage physical activity [17] and social cohesion [18], which support the improvement of cognitive functioning [19,20]. Additionally, greenspaces have a restorative value, as proposed by the Stress Reduction Theory (SRT) [21,22] and by the Attention Restoration Theory (ART) [23,24,25]. The SRT suggest that exposure to the natural environment and greenspace encourages positive emotions and positive change in physiological arousal, which preserves sustained attention [21,22]. Instead, according to ART, since natural environments are sources of fascination, being in contact with the natural environment stimulates the use of involuntary attention. This could be an efficient way to recover depleted attention resources. Few studies have focused on the association between greenspace and attention or cognitive functioning in general. In a systematic review, de Keijzer et al. (2016) selected 13 studies on the relationship between long-term greenness exposure and cognition across the life course. Six studies focused on children, three on adults, and four on older adults. Studies on children highlighted the beneficial role of greenspace exposure and cognitive abilities, such as attention and working memory [26]. Moreover, studies reported a positive association of this exposure with cognitive function in adults as well [26]. Concerning older adults, results for associations between greenspace and cognitive functioning were limited and inconsistent [26]. Therefore, the authors concluded that evidence on the association between greenspace exposure and cognitive functioning were still inadequate but suggestive for potential association and are thus worthy of investigation. Since the publication of the aforementioned systematic review, several studies investigating the same association were published.

The overarching goal of this study was to systematically evaluate the body of evidence on the association between greenspace exposure and cognitive functioning. Specific aims were as follows: (a) to summarize studies on the topic including only objective measures of greenspace exposure and cognitive functioning; (b) to evaluate the beneficial role of greenspace in different age groups and for specific cognitive domains (e.g., attention, intelligent quotient (IQ), or global cognition); (c) to provide an explorative overview of intervening variables that could account for mediation or moderation effects on the association between greenspace exposure and cognitive functioning.

## 2. Methods

### 2.1. Eligibility Criteria

The PECO method [27] was used to define the selection criteria for the suitable studies: (a) P (Participants): no age, sex, or health condition restrictions were applied; (b) E (Exposure): long exposure to greenspace, assessed with objective measures; (c) C (Comparison): no comparison; (d) O (Outcome): global cognition, memory, attention/executive functions, visuospatial abilities, and language, as outcome, assessed with objective measures. Moreover, we only included original articles that were written in English and were based on human studies without any limitation with regards to the year of publication. Case studies, editorials, review articles, and conference abstracts were excluded from our review.

### 2.2. Search Strategy and Study Selection

The Preferred Reporting Items for Systematic Reviews and Meta-Analyses (PRISMA) guidelines [28] was applied for reporting results of the present review. We queried PsychINFO, Scopus, and PubMed to search for the eligible studies. PsychINFO is considered the most used abstracts database of psychological sciences. Scopus is identified as a well-used electronic database of peer-reviewed research in several fields, such as medicine and life sciences. Instead, PubMed is considered to be an interface for searching MEDLINE, the most-used electronic database for health sciences.

The search strategy was defined using the following syntax terms based on title, abstract, and keywords: “greenness” OR “greenspace(s)” OR “urban forestry” AND “cogn *” OR “memory” OR “attent *” OR “lang *” OR “visuospatial” OR “exec *”. The search was conducted on 31 January 2022. The search syntax terms were adjusted to fit each database as presented in the Supplementary Materials (Table S1). Other studies were added by checking the reference list of the selected studies. From the resulting records, duplications were excluded. The articles were screened for the eligibility in three steps. Firstly, articles were screened for title and then for abstract. The final screening step was performed for the full text. The selected articles were consistent with the eligibility criteria.

### 2.3. Data Extraction and Manipulation

A datasheet from the electronic database was carried out by the authors to manage the large body of articles using R package “xlsx” [29,30]. For each selected study, the following information was extracted: authors, year, country, study design, study population, sample population, level of greenspace, greenspace indicator(s), outcome, outcome assessment, covariates, mediation and moderation variables, statistical analyses, and main study results. Associations found in each study included in the final dataset were assessed according to the Bayesian average method. It was used to avoid bias due to the discrepancy in the number of analyses performed in the included studies (e.g., using the percentage, the number of significant associations should be divided by the total number of analyses and multiplied by one hundred, and if ten analyses were performed and 5 significant associations were found, it should be attributed 50% of the associations to that study, but if 1 analysis was performed and 1 significant association was found, it should be attributed 100%). The Bayesian average was estimated considering (a) p (proportion of the significant analyses performed for each study), (b) c (the 25th percentile of the distribution of the number of analyses performed for each study), (c) m (mean of p), and (d) n (the total number of analyses performed for each study). The following formula was used:

(p × n + c × m)/(n + c)

Each study was classified as reporting a small association if the Bayesian average ranged between 0 and 0.33, medium association if it ranged between 0.33 and 0.66, and strong association if it ranged from 0.66 and 1.00.

### 2.4. Quality Assessment

Each article was evaluated for its quality. The score was based on 11 criteria for quality assessment of the studies that were adopted from similar previous systematic reviews of the health impacts of long-term exposure to green space (Table S2, Supplementary Materials) [8,26,31]. The quality score included a range from 0 to 1 for eight items of the checklist and from 0 to 2 for three items. The highest total score possible was 14; the total score for each article was converted to a percentage: it was divided by the maximum total score possible, and the result was multiplied by one hundred. The range quality was then labeled as excellent quality (score ≥ 81%), good quality (score between 61% and 80%), fair quality (score between 41% and 60%), poor quality (score between 21% and 40%), and very poor quality (score ≤ 20%). Two authors (ER and AOC) independently provided their quality score on each article. A third author (GSp) provided his quality score in case of disagreement. Cohen’s Kappa was then used to obtain a measure of inter-rater agreement. A value of K = 0.83 was found, thus indicating a good agreement between the two raters [32].

## 3. Results

### 3.1. Study Selection

**Figure 1:**
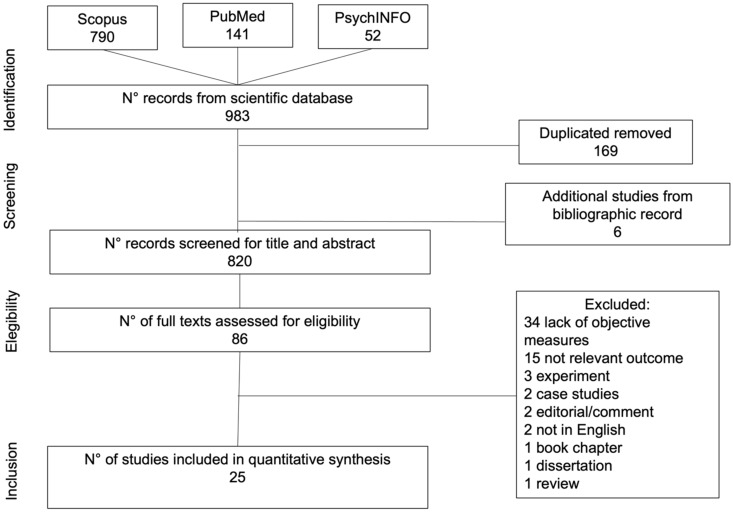
Flowchart for selection process of articles.

Figure 1 presents the selection process of the articles. Initially, a total of 983 articles were found based on our systematic research, of which 169 studies were eliminated because they were duplicated. In addition, six studies from the references of the selected studies were identified. A further 734 articles were excluded after screening the titles and abstracts, because they did not meet our selection criteria. The remaining 86 articles were screened by full-text and 61 articles were excluded, of which 34 did not use objective measures of greenspace and/or cognitive functioning, 15 did not include relevant outcome for the present review, 3 were experiments, 2 were not written in English, 2 were case studies, 2 were editorials/commentaries, 1 was a review, 1 was a book chapter, and 1 was a dissertation. Therefore, 25 articles met the selection criteria and were included in this systematic review.

### 3.2. Study Characteristics

Table 1 shows the main characteristics of the selected studies. The most of studies (N = 27) were published after 2016, when the de Keijzer’s review was conducted. Among the selected articles, 6 studies were longitudinal [33,34,35,36,37,38] and 19 were cross-sectional studies [39,40,41,42,43,44,45,46,47,48,49,50,51,52,53,54,55,56,57].

The selected studies were from the USA (N = 8) [37,45,48,49,50,52,53,55], Spain (N = 5) [33,34,35,36,42], England (N = 2) (Flouri et al., 2019; Lega et al., 2021), China (N = 3), Australia (N = 2) [40,41], Canada (N = 1) [46], New Zealand (N = 1) [54], and Bulgaria (N = 1) [43]. One study collected data from four European countries: Spain, England, Lithuania, and the Netherlands [57]. Fifteen studies were conducted among children [33,34,35,37,39,41,44,45,48,49,50,52,53,54,55], six on adults [40,42,43,46,51,57], and four among older adults [36,38,47,56]. The selected studies mainly assessed exposure to greenspace across buffers with a radius ranging from 25 m to 1000 m. Most of the studies (N = 17) [33,34,35,36,37,38,41,42,43,46,47,49,51,52,55,56,57] used the Normalized Differences Vegetation Index (NDVI). Two studies used also the Enhanced Vegetation Index (EVI) and Vegetation Continuous Field (VCF) [34,36], the additional indexes that, respectively, measure vegetation and tree cover [58,59]. Ten studies [39,40,44,45,48,49,50,52,53,54] also used other indicators of greenspace exposure as data from Multiple Environmental Deprivation Index (MEDIx), tree canopy cover, grass/shrub cover, and average percent impervious surfaces, and one study used the percentage of time spent in a greenspace. A deeper description of the characteristics of greenspace exposure assessment is in Table S3, Supplementary Materials.

Evaluated outcomes varied among the studies across the age groups: attention and executive functions among children, adolescents, and adults (N = 6) [33,34,35,39,46,57], memory among children and adults (N = 3) [40,44,51], global cognition among children, adults, and older adults (N = 8) [36,38,42,43,47,54,56,60], and academic achievement among children (N = 8) [41,45,48,49,50,52,53,55].

All studies, except for four [35,45,53,54], used more than three confounders in order to adjust their models. The most applied covariates were age, sex, and socioeconomic status. In some of studies on children, models were adjusted also for other confounders such as maternal or paternal education, maternal cognitive functioning, and maternal smoking during pregnancy. Among the studies on adults, smoking, alcohol, blood pressure, waist circumference, marital status, and employment were used as covariates as well. Instead, in the older adults’ studies, the models were adjusted also for financial support, physical activity, and social and leisure activities. Twelve studies [33,36,37,41,43,44,47,48,51,53,56,57] took into account mediation variables and effect modifiers.

Table 2 shows selected studies classified according to the Bayesian average method. Eight studies [39,42,45,46,47,53,57,60] showed a small association between greenspace exposure and cognitive functioning, eleven studies indicated medium association [33,36,38,41,44,48,49,50,51,54,56], and six studies revealed strong association [34,35,40,43,52,55].

### 3.3. Study Findings

#### 3.3.1. Children and Adolescents

Fifteen studies investigated the association between exposure to green space at home, school, and/or on the commuting route between home and school and cognitive development in children. The studies were conducted in Europe (N = 5), America (N = 8), and Oceania (N = 2). Fourteen studies were classified as good quality, and one study as fair quality. Among the overall analyses and according to the Bayesian average, four studies [37,39,45,53] showed small association, seven studies [33,41,44,48,49,50,52] displayed medium association, and four studies [34,35,52,55] were evaluated as having a strong association (Table 3).

Eight of the selected studies on children considered academic achievement as an outcome; among these, three cross-sectional studies [41,52,55] found that a higher level of greenness surrounding primary schools was associated with higher academic achievements among schoolchildren. Specifically, Claesen et al. (2021) examined mean academic score in primary schools in Australia and found a significant and positive association between NDVI levels and the domains of reading, numeracy, and grammar/pronunciation. Wu et al. (2014) found a significant association between greenness of the school in spring and academic performances in math and English among children in elementary school in Massachusetts. Leung et al. (2017), as well, showed that the associations were positive for greenness around the school in Massachusetts and academic performances measured by composite performance index and percentage of students who scored as “proficient and higher”. Another two cross-sectional studies [49,50] highlighted that a higher percentage of tree cover in school surrounding was associated with better performance in math and reading tests. In one of these [49] conducted in Washington, greenness, in a buffer of 250 m, was associated with reading and math scores as well. Instead, Kuo et al. (2018) found a positive and significant association between school trees and math scores, but not for reading scores in public schools of Chicago. In contrast, Hodson and Sander (2017) reported an association between tree cover and reading performances in a sample of primary schools in Minnesota. For academic achievement as the outcome, Sivarajah et al. (2018) did not find any association between performance at elementary schools in Toronto (N = 387) and tree cover.

Two studies considered global cognition in children as the outcome. Especially, Jimenez et al. (2022), among the assessed cognitive domains, found an association between NDVI and visual memory in Massachusetts. On the contrary, Ward et al. (2016) did not find any association between time spent in greenspace and global cognition in children of Auckland [54].

Three longitudinal studies and two cross-sectional studies found an association between greenspace exposure and attention/executive functions and memory among children. In their Spanish study, Dadvand et al. (2015) found an association between 12-months progress in working memory and attention and greenness within school, surrounding school, or total surrounding greenness; commuting greenness, instead, was only associated with 12-months progress in working memory, but there was no association between residential surrounding greenness and working memory or attention at baseline or progress. Moreover, Dadvand et al. (2018) found an association between surrounding greenness and volumes in brain regions related to working memory and inattentiveness. In another study, in two cohorts of children in Spain, exposure to residential greenspace, measured as average NDVI, was associated with lower inattentiveness [34]. However, the associations between residential surrounding tree cover (i.e., based on VCF) and inattentiveness were not statistically significant. Bijnens et al., (2021) found that an increase in total greenspace (within 2000 m) was association with a better performance in attention and executive functions tasks in Belgian adolescents. Especially, vegetation higher than 3 m (high green) was associated with a shorter reaction time in attentional tasks. Lastly, the cross-sectional study conducted by Flouri et al. (2017) reported a significant association between neighborhood greenspace and spatial working memory in children in England.

#### 3.3.2. Adults

Six studies investigated the association between residential greenness exposure and cognitive abilities among adults. All the studies were cross-sectional. The studies were conducted in Europe (N = 4), Oceania (N = 1), and North America (N = 1). Four studies were classified as good quality and two studies were classified as fair quality. Using the Bayesian average, three studies [42,46,57] were classified as small associations, one study [51] showed medium association, and two studies showed strong [40,43] association (Table 3).

Dzhambov et al. (2019), in a middle-aged population in Bulgaria, observed that living in neighborhoods with a higher ratio of greenspace (i.e., NDVI) was associated with better performance in general cognitive abilities. A cross-sectional study conducted in Spain did not find any association between residential surrounding greenness and global cognition, episodic memory, and executive functions [42]. Furthermore, another cross-sectional study in England [51] study reported a beneficial association of greenness surrounding home address on memory tasks. Specifically, residential surrounding greenness was significantly associated with forward digit span and total digit span, but there was no association with backward digit span. Concerning executive functions, in a sample of 1628 adults, an association between residential distance to natural outdoor environments and executive domains was found [57]. However, another study conducted in Canada by Hystad et al. (2019) did not find any associations between greenspace and executive functions among adults.

#### 3.3.3. Older Adults

Four studies evaluated the relationship of greenspace exposure and risk of cognitive decline in older adults. Two studies were longitudinal and two were cross-sectional. The studies were conducted in Europe (N = 4), Oceania (N = 1), and North America (N = 1). All the studies on older adults were classified as good quality. One study showed a small association [47], and three studies [36,38,56] displayed a medium association according to the Bayesian average (Table 3).

In their longitudinal study in China, Zhu et al. (2019) showed that an increase in residential greenness exposure was associated with a better performance in Mini-Mental State Examination (MMSE) and a highest-odds ratio developing some cognition impairments. In addition, there was an association between residential greenness exposure and changes in MMSE score in the longitudinal analysis. The association between residential greenness exposure and odds of cognitive impairment was also found in another study [56], particularly in older adults aged from 65 to 79 years.

De Keijzer et al. (2017), in their longitudinal study, found that higher levels of greenspace (i.e., NDVI, EVI) in a 500 m and 1000 m buffer around the residential address were associated with slower cognitive decline in global cognition, reasoning, and fluency in older Spanish adults. Similarly, Jin et al. (2021) found that the highest contemporaneous NDVI (defined as a single measure of NDVI) was associated with lower odds of cognitive impairment, but no significant association was found between annual average of NDVI and odds in cognitive impairment in older Chinese adults.

#### 3.3.4. Mediators and Effect Modifiers

Our reviewed studies considered the air pollution, stress, social interactions, blood pressure, physical activity, and obesity as potential mediators and sex, indicators of socioeconomic position learning opportunity index, and APOE ε4 as potential effect modifiers. Four studies tested the mediation role of air pollution. Specifically, Jimenez et al. (2022) reported a significant negative mediated effect of black carbon in the association between early childhood greenness and midchildhood cognitive development (except for verbal IQ). Dzhambov et al. (2019) did not find a mediating role of nitrogen dioxide in the association between residential surrounding greenness and cognitive abilities in adults. Dadvand et al. (2015) observed that the beneficial association of greenspace exposure with attention and working memory among children was partially mediated by reduction in TRAP. Furthermore, in another study by Cleasen et al., TRAP was reported to mediate the association between greenness around schools and academic achievement in terms of numeracy and grammar/punctuation [41]. Stress was evaluated as a mediator in the relationship between surrounding greenness and memory in only one study [51], which reported a partial mediation effect. Lastly, the mediation role of waist circumference, as an indicator of obesity, in the association between residential greenness and cognitive functions was found in a cross-sectional study among adults [43]. Other mediators were considered [36,37,43,57], such as social interaction/support/cohesion, blood pressure, and physical activity, but none of them showed a significant mediatory effect.

Four studies evaluated the effect modifiers. Flouri et al. (2019) investigated the modification of the association between greenness and cognitive functioning by the neighborhood deprivation and found that the association of greenness on spatial working memory did not change across different levels of neighborhood deprivation in a sample of children. In their study, Sivarajah et al. (2017) suggested that the association of tree cover with academic achievement changes across different levels of the learning opportunities index. Interaction between tree canopy cover and SES disadvantage in association with academic achievement was explored by Kuo et al. (2018). Their findings suggested that the association between school trees and academic achievement was modified by socioeconomic disadvantage (investigated by income and race/ethnicity). Lastly, Jin et al. (2021) found a significant interaction between residential greenness and AD Poligenetic Risk Score on cognitive functioning in older people. In addition, according to Zhu et al. (2020), the status of APOE ε4, considered to be a relevant risk factor in developing Alzheimer’s disease [61], was found to be a potential modifier of the association between greenspace exposure and cognitive impairment. Nevertheless, the interaction term between baseline annual average NDVI and APOE ε4 status on cognitive impairment was not significant.

## 4. Discussion

The purpose of this systematic review was to synthesize the available evidence on the association of greenspace exposure with cognitive function across the life course. Accordingly, we reviewed studies on this association across different age groups for different objective measures of greenspace exposure and cognitive domains (i.e., memory, attention, executive functions, visuospatial abilities, global cognition) and identified the reported potential mediators and modifiers of such associations.

The selected studies totaled 25. All the selected studies were published after 2016. A lot of studies on the beneficial role of the greenness exposure on the cognitive functioning were published over the past few years. This issue highlighted the need for an updated literature review. Moreover, differently from de Keijzer et al. (2016), all the selected studies used objective measures of greenspace exposure that are considered the better methods to explore the relationship between greenspace and health [14]. In addition, the selected studies were conducted mainly in Europe and North America: few studies were conducted in Asia and Oceania. Therefore, the selected studies were not conducted in many different climates and with different vegetation types. In addition, a lot of study were conducted especially in middle- and high-income countries.

The attempt to summarize findings on the association between greenspace exposure and cognitive functioning was difficult due to limitations of the available evidence, such as different study design, different number of analyses performed, and a great variety of predictors and outcomes. To overcome this, in our systematic review, we assessed each study based on the Bayesian average and each study was classified as small association, medium association, and strong association.

### 4.1. Age Groups

Among children, associations were found in attention/EF, memory, and academic achievement. This finding was consistent with previous reviews supporting the beneficial role of natural environment for schoolchildren [7,62]. For global cognition, the beneficial role of greenness exposure remains unclear. Within the adults’ age group, the trend is more blurred. Strong associations were found only for two of the four studies that investigated global cognition and memory. Therefore, among the selected evidence, all the studies investigating attention showed a small association. For older adults, few studies met our selection criteria, and all showed small or medium association between greenspace exposure and global cognition.

Overall, a general unclear trend on the relation between greenspace exposure and global cognition during the lifespan emerged, with studies on children and older adults lacking full associations for global cognition. A positive trend was found for attention and executive functions, which is in agreement with the ART. This trend was detectable exclusively across the children’s and adults’ age group. This finding was consistent with results from Jimenez et al. (2021). Indeed, their review suggested that the impact of greenspace exposure on cognitive functioning among adults was comparable with results obtained from children’s studies. A similar trend was not detectable across the older adults’ group due to a lack of studies on attention and executive functions in aging, even though recent studies suggested that the presence of greenspace could reduce the risk of developing dementia [16]. Furthermore, several studies were carried out on samples composed of children and few studies were available on the adults’ and older adults’ age groups. Nevertheless, consistent with the available literature [26,63,64], more studies on adults and older adults could be useful to explore the role of environment, especially of greenness exposure, in cognitively healthy aging and age-related cognitive decline.

### 4.2. Study Design

The present systematic review included 25 studies, and more than half of them were cross-sectional. Although cross-sectional designs are commonly adopted to explore the association between variables, their use leads to some methodological limits. The cross-sectional study implicates that all variables are assessed simultaneously. For this reason, the cross-sectional study has a predictive limitation, and no evidence on causal relationship between the variables could be deduced [65]. Longitudinal studies, instead, could overcome this limitation and provide reliable knowledge about the predictive conclusions.

### 4.3. Greenspace Exposure

In order to assess greenspace exposure and contact with greenspace, different methods are available. Surrounding greenness is the most-used. Almost all selected studies used the surrounding greenness to take account of greenness exposure. The most-used indicator of surrounding greenness was the NDVI. The NDVI is an efficient metric used to assess the presence of vegetation and is delivered from satellite images which quantify vegetation studying the difference between near-infrared vegetation minus visible radiation divided by near-infrared radiation plus visible radiation. It ranges from minus −1 to +1, with 0 indicating the absence of vegetation. Instead, if the index is close to +1, it indicates the presence of high density of green leaves [66]. The use of the NDVI allows a comparison among different studies. Nevertheless, the NDVI cannot evaluate the quality, typology, and biodiversity of greenspace and does not give information about structured greenspaces, such as parks, and unstructured vegetation, such as trees in the streets or yards [14]. To overcome the limits of NDVI, other indicators were used by the selected studies, such as EVI and VCF, two additional indexes useful, respectively, in monitoring vegetation and in measuring ground cover [58,59] and tree canopy cover, grass, and shrub. Using various indicators could make the comparison among different studies difficult and, as suggested by other authors [8,14], standardized tools to assess greenspace exposure could be useful in this research field. In addition, we detected that several selected studies used surrounding greenness focused on exposure at the home address or surrounding school, overlooking the exposure that can occur in other microenvironments such as workplace or commuting route, as suggested by a previous review [26]. Furthermore, most of the reviewed studies (except one: Hystad et al. (2019)) did not take into account changes in residential address.

Several studies measured greenness exposure within a buffer from 30 m to 5000 m, but it is not clear what buffer distance could be more usefully assessed [14]. Indeed, despite a large agreement on the use of specific buffer for NDVI (i.e., 100 m, 150 m, 300 m), official guidelines are still lacking. Best practices from previous studies should be considered in order to clarify which areas and buffer distances could be advantageous to measure [14,67,68].

Physical access to greenspace is a valid method to assess contact with greenspace as well. Few selected studies used it and quantified the distance between the address and the closest greenspace.

Visual access to greenspace and use of greenspace were never considered in our selected studies.

Lastly, in line with previous studies [26,64], we detected a few considerations of quality of greenspace that may play a key role in the association between greenspace and cognitive functioning, such as aesthetics, walkability, safety, biodiversity, and organized social activities [69].

### 4.4. Cognitive Functioning

Accounting for cognitive domains considered in the selected studies, cognitive domains were differentially measured through the age groups (i.e., children, adults, and older adults). Cognitive development in children was assessed considering different outcomes (e.g., attention/EF, memory, global cognition, academic achievement). All outcomes were assessed with a standardized cognitive test. Academic achievement was assessed with measures of school performance that may be influenced by other cognitive domains such as attention and executive functions.

Cognitive functioning in adults was assessed with standardized tests as well for each of the cognitive domains, such as the free recall test and S-words test. Instead, to evaluate cognitive functioning in older adults, the reviewed studies used a single screening test for global cognition (i.e., MMSE), making it difficult to have a clear overview for each specific cognitive domain. As suggested by ART, some proprieties of greenspace could be related with specific cognitive domain, not measurable with a single screening test. To overcome this, the Montreal Cognitive Assessment might be used, the most comprehensive available single screening test [70,71] to explore each cognitive domain separately. Therefore, well-established best practices to assess cognitive functioning among older adults could be used. This could provide a clear overview for each specific cognitive domain in older people, including spatial memory and orientation, which are sensitive to age and familiarity for places [72,73]. Lastly, computerized tools and evaluation by healthcare professional may provide a more accurate assessment of cognitive functioning across the age groups.

### 4.5. Role of Mediators and Modifiers

Few studies included in the present review explored the mediators of the association between greenspace exposure and cognitive function. TRAP, stress, and obesity were found to be potential mediators of this association; however, these observations were limited and in some cases were inconsistent. For example, the findings about the role of air pollution in the association between greenspace exposure and cognitive functioning were not consistent [37,43]. The mediation role of TRAP was not clear as well, but some studies highlighted that the association between greenspace exposure and cognitive functioning could be mediated by a reduction of TRAP in green areas [33,41].

Little evidence on the role of moderating variables was available as well. In spite of that, some studies suggested the modifying role of learning opportunities and socioeconomic status in the association between greenspace exposure and cognitive functioning [48,53]. No study investigated perceived restoration in this association. According to ART, it could be usefully introduced it in future models.

## 5. Limitations

The present review has some limitations. The variety of outcomes did not allow us to perform a formal meta-analysis. In addition, due to our limiting selection criteria, we excluded many studies evaluating the role of green exposure on cognitive functioning, since they used subjective measures of assessment.

## 6. Conclusions

The aim of the present work was to systematically review and summarize the available studies on the beneficial role of greenspace exposure on cognitive functioning. We found a limited number of available studies and most of them were cross-sectional. Cognitive domains were evaluated with different tools through the age groups and few studies explored intervening variables that could mediate or moderate the association between greenspace exposure and cognitive functioning. The available evidence is still limited, especially for adults and the elderly, but still is suggestive for a beneficial association between exposure to greenspace and cognitive function across the life-course. Further research could benefit from (a) longitudinal designs; (b) further focus on middle-aged and older adults; (c) the use of well-established practices to assess cognition; (d) the assessment of quality of greenspace; (e) the consideration of different climates with different vegetation types and in under-represented regions, especially in low- and middle-income countries; (f) a deeper investigation of mechanisms and potential effect modifiers.

## Figures and Tables

**Table 1:** Main characteristics of the studies.

Authors, Year	Study Design	Country	Continent	Study Population	Sample Population	Level of Greenspace	Greenspace Indicator	Outcome	Outcome Assessment	Covariates	Mediation and Effect Modifiers	Statistical Analyses	Main Result
Claesen et al., 2021 [41]	Cross-sectional	Australia	Oceania	Children	851 primary schools	School surrounding greenness	NDVI	Academic achievement	NAPLAN scores	School sector NAPLAN test format Number of girls’ enrolments Number of boys’ enrolments FTE of enrolled students FTE of teaching staff enrolments area Level of socioeconomic status for each school	Mediating role of TRAP	Generalized linear models	Association between NDVI and reading scores for students in years 3 and 5 in all buffers (except 2000 m, Year 3) Association between NDVI and numeracy scores in years 3 and 5 for all buffers and grammar/punctuation scores in year 5 for all buffers Inverse associations between NDVI and spelling scores in years 3 and 5 for all buffers (except the school polygon) No association between NDVI and writing scores
Dadvand et al., 2015 [33]	Longitudinal	Spain	Europe	Children	2593 children	Residential surrounding greenness Commuting greenness School greenness	NDVI	Attention/EF	N-back task ANT	Age Sex SES at individual level SES at area level	Mediating role of TRAP	Linear mixed-effect models	Association between 12 mo progress in WM/superior WM/attention and greenness within school/surrounding school Association between 12 mo progress in WM/superior WM/attention and total surrounding greenness Association between 12 mo progress in WM and commuting greenness No association between residential surrounding greenness and WM/superior WM/attention at baseline or progress
Dadvand et al., 2017 [34]	Longitudinal	Spain	Europe	Children	1527 children	Residential surrounding greenness	NDVI VFC	Attention/EF	K-CPT ANT	Age Sex Term birth Maternal cognitive performance Maternal smoking during pregnancy Exposure to environmental tobacco smoke SES at individual level SES at area level Urban vulnerability index	/	Mixed-effect models	Increases in residential surrounding greenness (NDVI) were associated with lower K-CPT omission and HRT-SE at 4–5 y and lower ANT HRT-SE at 7 y No association between K-CPT commission errors and ANT omission or commission errors
Dadvand et al., 2018 [35]	Longitudinal	Spain	Europe	Children	253 children	Residential surrounding greenness	NDVI	Attention/EF	3D MRIs ANT 2-back tasks 3-back tasks	Maternal education SES	/	Linear mixed-effects model	Association between residential surrounding greenness and volumes in several brain regions Association between some of these regions and WM or superior WM or inattentiveness
Flouri et al., 2019 [44]	Cross-sectional	England	Europe	Children	4758 children	Neighborhood greenspace	Data from Multiple Environmental Deprivation Index (MEDIx)	Memory	CANTAB SWM task	SES Neighborhood history Neighborhood deprivation Gender Age	Neighborhood greenspace * Neighborhood deprivation	Multilevel linear model	Association between neighborhood greenspace and SWM (b = 0.793; SE = 0.384; 95%; CI: −1.545, −0.041)
Hodson t al., 2017 [45]	Cross-sectional	USA	America	Children	222 primary schools	School greenness	Average percent canopy cover Average percent impervious surfaces Grass/Shrub cover	Academic achievement	MCA	SES ELL Lunch	/	Ordinary least squares regression models	Association between canopy and reading (b = 0.26846; t-value = 2.572) No association between canopy and mathematics score No association between grass or shrub and reading or math score
Jimenez et al., 2022 [37]	Longitudinal	USA	America	Children	857 mother–child pairs	Residential surrounding greenness	NDVI	Global cognition	PPVT-III WRAVMA WRAML2 KBIT-2	Sex Race Age Mother’s intelligence Parent’s education Annual household income at enrollment Neighborhood median annual income Neighborhood population density	Air PollutionPhysical Activity	Generalized additive models	Greenness at early childhood was associated with visual memory (0.76; 95%; CI: 0.21–1.32)
Kuo et al., 2018 [48]	Cross-sectional	USA	America	Children	318 public schools	School and neighborhood greenness	Tree canopy cover Grass/shrub cover	Academic achievement	ISAT assessment	Disadvantage Bilingual Number of students % female pupil/teacher ratio	School greenness * Disadvantage Neighborhood greenness * Disadvantage	Generalized linear models	Association between school trees and math scores (b = 0.22; SE = 0.10) Marginally significant association between school tree and reading scores No association between neighborhood trees and math scores/reading scores
Kuo et al., 2021 [49]	Cross-sectional	USA	America	Children	450 public schools	School greenness	NDVI Tree canopy cover	Academic achievement	Washington Measurements of Student Progress Assessment	Race/ethnicity Poverty Transitional bilingual status Sex Special education Section 504 status Students per teacher Average years of educational experience among teachers The percentage of teachers with master’s degrees School enrollment and location (urban, suburban, or rural)	/	Multivariate analyses	Tree canopy within 250 m of a school predicted better performance in both reading (coeff = 0.117, *p* = 0.000) and math (coeff = 0.134, *p* = 0.134), as well total greenness within 250 m (reading coeff = 0.131, *p* = 0.036; math coeff = 0.179, *p* = 0–0.39), and tree canopy within 1000 m (reading coeff = 0.068, *p* = 0.017; math coeff = 0.079, *p* = 0–0.47). At the 1000 m buffer size, total greenness does not predict achievement Tree canopy predict achievement when total greenness was controlled (reading coeff = 0.161, *p* = 0.001; math coeff = 0.153, *p* = 0.020) Tree canopy at 250 m was significant for reading and math even when tree cover at 1000 m was controlled (reading coeff = 0.174, *p* = 0.001; math coeff = 0.187, *p* = 0.012)
Kweon et al., 2017 [50]	Cross-sectional	USA	America	Children	219 public elementary and secondary schools and learning center	School greenness	Land cover variables	Academic achievement	DC Comprehensive Assessment System	SES Enrollment Student/Teacher Ratio Race/Ethnicity	/	Linear regression analyses	Association between trees (%) and mathematics (b = 0.23; *p* = 0.005)/reading tests (b = 0.22; *p* = 0.006). No association between grass or shrubs (%) and reading/mathematics
Leung et al., 2019 [52]	Cross-sectional	USA	America	Children	2749 children	Greenness surrounding school	NDVI Green land use	Academic achievement	MCAS test	Sex Student-teacher ratio Financial status Language ability Race and ethnicity	/	Generalized linear mixed models	Except the result of green land use of ELA in 250 m buffer, associations were all significantly (*p* < 0.05) positive for surrounding greenness and academic performance (AP%/CPI)
Ward et al., 2016 [54]	Cross-sectional	New Zealand	Oceania	Children	108 children	Greenspace	Time spent in GS	Global cognition	CNS-VS	Sex Age School	/	Generalized linear mixed models	Significant results not found
Wu et al., 2014 [55]	Cross-sectional	USA	America	Children	905 schools	Greenness of school surrounding	NDVI	Academic achievement	MCAS	Gender Race English as a second language Family income level Student/teacher ratio School attendance Country of schools	/	Spatial Generalized linear mixed models	Significant association (*p* < 0.01) between surrounding greenness in March and academic achievement in English and math for all buffers. Considering July and October, students with higher exposure to greenness for the balance of the year (even in summer) show better academic performance, too, with most of the estimates showing statistically significant results (p. 0.05)
Sivarajah et al., 2018 [53]	Cross-sectional	USA	America	Children	387 elementary schools	Vegetation around school	Total land area (m^2^) Total soft surface (m^2^) Tree canopy cover (m^2^) Percentage tree cover	Academic achievement	Student performance	Socio-demographic Economic factors	tree cover * LOI	Generalized Linear Models	Significant results not found
Bijnens et al., 2022 [39]	Cross-sectional	Belgium	Europe	Adolescents	596 adolescents	Residential surrounding greenspace School surrounding greenspace Proximity to accessible greenspace	Land cover data from the Agency for Geographic Information Flanders	Attention/EF	Stroop Test Continuous Performance Test	Age Sex Education level mother Area deprivation index		Multiple linear regressionLogistic regression model	The association was found between the higher total and high greenspace (at 2000 m radius) with a shorter reaction time on Stroop Test and the CPT. An increase of 13% in greenspace (within a 2000 m radius) is associated with a 35% lower risk of a mean reaction time longer than 536 ms on the Stroop Test and with a 24% lower risk of a mean reaction time longer than 1476 ms on the CPT
Cerin et al., 2021 [40]	Cross-sectional	Australia	Oceania	Adults	4141 adults	Parkland in residential buffer	Percentage of parkland in residential buffer	Memory	CVLT SDMT	Age Sex English-speaking background Educational attainment Population density Percentage of commercial land use Land-use mix (five noncommercial land uses) Area-level IRSAD Residential self-selection related to recreational facilities	/	Generalized additive mixed models	The percentage of parkland in residential buffer was associated with better performance in memory and processing speed in total and direct-effect model
Lega et al., 2021 [51]	Cross-sectional	England	Europe	Adults	185 adults	Residential surrounding greenness	NDVI	Memory	FDS BDS TDS	Gender Educational level Deprivation Frequency of visits to natural environments Age	Mediating role of stress	Linear univariate regression	Association between surrounding greenness and FDS (b = 0.45, 95% CI: 12.59, 21.10) Association between surrounding greenness and TDS (b = 0.34, 95% CI: 10.50, 26.12) No association between surrounding greenness and BDS
Dzhambov et al., 2019 [43]	Cross-sectional	Bulgaria	Europe	Adults	111 adults	Residential surrounding greenness	NDVI	Global cognition	CERAD-NB MoCA	Sex Age Education Smoking Alcohol consumption Waist circumference Blood pressure Road traffic day-evening-night noise	Mediating role of waist circumference, systolic blood pressure, total cholesterol, air pollution, glucose, NO_2_, and L_den_	Multivariate linear regression models	Association between NDVI and CERAD-NB and MoCA, especially for NDVI 100 m
Zijlema et al., 2017 [57]	Cross-sectional	Spain Lithuania Netherlands England	Europe	Adults	1628 adults	Residential surrounding greenness	NDVI Distance to NOE	Attention/EF	CTT	Age Sex Educational level Neighborhood socioeconomic status Time spent away from home CTT test quality	Mediating role of physical activity, social interaction, loneliness, neighborhood social cohesion, perceived mental health, traffic noise, worry about air pollution	Linear and logistic multilevel models	Association between residential distance to NOE (per 100 m) and CTT time (b = 1.50; 95%, CI: 0.13–2.89) No association between other indicators of NOE and CTT (time or errors)
Hystad et al., 2019 [46]	Cross-sectional	Canada	America	Adults	6658 adults	Residential surrounding greenness	NDVI	Attention/EF	Paired associated learning Reaction time Verbal and numeric reasoning	Year and month of completion of baseline questionnaire Age Sex at birth (male/female) Household income Education level White/nonwhite Marital status Population density	/	Linear and logistic regression models	Significant results not found
Crous-Bou et al., 2020 [42]	Cross-sectional	Spain	Europe	Adults	958 adults	Residential surrounding greenness	NDVI	Global cognition	MBT WAIS-IV PACC	Age Gender Years of education	/	General linear models	Significant results not found
De Keijzer et al., 2017 [36]	Longitudinal	Spain	Europe	Older adults	6506 older adults	Residential surrounding greenness	NDVI EVI	Global cognition	Alice Heim 4 S-words test Animal names test Free recall test	Age Gender Ethnicity Alcohol use Diet Smoking Education IMD IMD employment SES Socioeconomic status Employment grade	Mediation role of physical activities, air pollution and social support	Mixed-effects model with repeated measures	An IQR increase in NDVI in a 500 m buffer was associated with a difference in the global cognition score of 0.020 (95% CI: 0.003, 0.037) over 10 years An IQR increase in NDVI in the 500 m buffer was associated with a difference in the reasoning z-score of 0.022 (95% CI: 0.007, 0.038) and with a difference of 0.021 (95% CI: 0.002, 0.040) in the fluency z-score over 10 years A positive baseline association between residential surrounding greenness and reasoning (b: 0.021; 95% CI: 0.003, 0.038)
Jin et al., 2021 [47]	Cross-sectional	China	Asia	Older adults	1349 older adults	Residential surrounding greenness	NDVI	Global cognition	Chinese version of MMSE	Smoking Drinking Physical activities Dietary diversity ADL Leisure activity score Seven kinds of self-reported disease (diabetes, heart disease, stroke, hypertension, chronic obstructive pulmonary disease, tuberculosis, and cancer)	Interaction between NDVI and AD-PRS on cognitive function	Multivariate logistic regression Linear regression model	Highest contemporaneous NDVI was associated with lower odds of cognitive impairment (Quartile 3: OR: 0.49, 95% CI: 0.31, 0.80, Quartile 4: OR: 0.62, 95% CI: 0.38, 0.99) 0.1-unit of contemporaneous average NDVI was associated with 9% lower odds (95% CI: 0.85, 0.99) of cognitive impairment and 0.28-point higher MMSE score (95% CI: 0.01, 0.56) No significant association was found between annual average of NDVI and cognitive impairment or MMSE
Zhu et al., 2019 [38]	Longitudinal	China	Asia	Older adults	19,726; 38,327 older adults	Residential surrounding greenness	NDVI	Global cognition	MMSE	Age Gender Ethnicity Marital status Urban/rural residence Education Occupation Financial support Social and leisure activity Smoking status Alcohol consumption Physical activity Time to reflect the number of years for each follow-up	/	Linear regressionLogistic regression Linear mixed-effects regression Mixed-effects logistic regression models	A 0.1-unit increase in NDVI was associated with a 0.23-point increase in MMSE score (95% CI 0.16 to 0.29) and an OR of 0.94 (95% CI 0.92 to 0.96) of having cognition impairment Participants living in areas with a decrease in greenness had an OR of 1.25 (95% CI 1.18 to 1.34) of a decrease in MMSE, and an OR of 0.90 (95% CI 0.84 to 0.96) of an increase in MMSE in the longitudinal analysis There was a significantly weak association (coefficient 0.069, 95% CI 0.0048 to 0.13) between NDVI and changes in MMSE
Zhu et al., 2020 [39]	Cross-sectional	China	Asia	Older adults	6994 older adults	Residential surrounding greenness	NDVI	Global cognition	MMSE	Age Gender Ethnicity Marital status Urban/rural residence Education Occupation Financial support Social and leisure activity Smoking status Alcohol consumption Physical activity	Moderation role of APOE	Generalized estimating equations	Older adults living in the highest quartile had 15% (95% CI: 0.75, 0.97) lower odds of cognitive impairment The association between residential greenness and cognitive function also differed by the age group The effect was significant only among the people aged 65 to 79 years (OR of the highest quartile of NDVI: 0.76, 95% CI: 0.62, 0.93)

Note: NDVI = Normalized Difference Vegetation Index; IQ = Intelligence Quotient; WISC III = Wechsler Intelligence Scale for Children-III; CI = Confidence Interval; IQR = Interquartile Range; NAPLAN score = National Assessment Program—Literacy and Numeracy score; FTE = Full Time Equivalent; TRAP = Traffic Related Air Pollution; ANT = Attentional Network Task; WM = Working Memory; VFC = Vegetation Continuous Field; K-CPT = Conners’ Kiddie Continuous Performance Test; SES = Socio-Economic Status; HRT-SE = Hit Reaction Time Standard Error; 3D-MRI = Three dimensional Magnetic Resonance Imaging; MEDIx = Multiple Environmental Deprivation Index; CANTAB = Cambridge Neuropsychological Test Automated Battery SWM task = Spatial Working Memory task; MCA = Minnesota Comprehensive Assessment; ELL = English language learners; PPVT-III = Peabody Picture Vocabulary Test; WEAVMA = Wide Range Assessment of Visual-Motor Abilities; WRAML2 = Wide Range Assessment of Memory and Learning; KBIT-2 = Kaufman Brief Intelligence Test; ISAT = Illinois State Board of Education’s Illinois Standardized Assessment Test; DC = District of Columbia; Kedi-WISC = Korean Educational Development Institute-Wechsler Intelligence Scale for Children; ETS = Exposure to Environmental Tobacco Smoke; NO_2_ = Nitrogen Dioxide; MCAS = Massachusetts Comprehensive Assessment System; AP = Proficient and Higher; CPI = Composite Performance Index; ELA = English Language Arts; WIPPSI-R = Wechsler Preschool and Primary Scale of Intelligence-Revised; WISC IV = Wechsler Intelligence Scale for Children-IV; WAIS IV = Wechsler Adults Intelligence Scale-IV; GS = Greenspace; CNS-VS = CNS visual signs; LOI = Learning Opportunity Index; CPT = Continuous Performance Test; CVLT = California Verbal Learning Test; SDMT = Symbol-Digit Modalities Test; FDS = Forward Digit Span; BDS = Backward Digit Span; TDS = Total Digit Span; CERAD-NB = Consortium to Establish a Registry for Alzheimer’s Disease Neuropsychological Battery; MoCA = Montreal Cognitive Assessment; Lden = Road traffic day-evening-night noise; NOE = Natural Outdoor Environment; CTT = Color Trails Test; MBT = Memory Binding Test; PACC = Preclinical Alzheimer Cognitive Composite; EVI = Enhanced Vegetation Index; IMD = Index of Multiple Deprivation; MMSE = Mini Mental State Examination; OR = Odds Ratio; AD-PRS = Alzheimer Disease Polygenic Risk Score; APOE = Apolipoprotein E.

**Table 2:** Associations between greenness and cognitive functions classified according to the Bayesian average method.

Authors, Year	Significant Result	Total Number of Results	*p*	*n*	Bayes Average	Association
Claesen et al., 2021 [41]	32	50	0.64	50	0.63	Medium
Dadvand et al., 2015 [33]	10	30	0.33	30	0.35	Medium
Dadvand et al., 2017 [34]	28	36	0.78	36	0.75	Strong
Dadvand et al., 2018 [35]	7	9	0.78	9	0.68	Strong
Flouri et al., 2019 [44]	1	1	1.00	1	0.58	Medium
Hodson et al., 2017 [45]	1	6	0.17	6	0.29	Small
Jimenez et al., 2022 [37]	2	8	0.25	8	0.32	Small
Kuo et al., 2018 [48]	1	4	0.25	4	0.36	Medium
Kuo et al., 2021 [49]	10	16	0.63	16	0.59	Medium
Kweon et al., 2017 [50]	2	4	0.50	4	0.49	Medium
Leung et al., 2019 [52]	31	32	0.97	32	0.91	Strong
Ward et al., 2016 [54]	0	1	0.00	1	0.38	Medium
Wu et al., 2014 [55]	20	24	0.83	24	0.78	Strong
Sivarajah et al., 2018 [53]	0	4	0.00	4	0.24	Small
Bijnens et al., 2022 [39]	5	36	0.14	36	0.17	Small
Cerin et al., 2021 [40]	4	4	1.00	4	0.74	Strong
Lega et al., 2021 [51]	2	3	0.67	3	0.55	Medium
Dzhambov et al., 2019 [43]	10	10	1.00	10	0.85	Strong
Zijlema et al., 2017 [57]	1	5	0.20	5	0.32	Small
Hystad et al., 2019 [46]	0	3	0.00	3	0.27	Small
Crous-Bou et al., 2021 [42]	0	3	0.00	3	0.27	Small
De Keijzer et al., 2017 [36]	8	16	0.50	16	0.49	Medium
Jin et al., 2021 [47]	4	16	0.25	16	0.29	Small
Zhu et al., 2019 [38]	6	16	0.38	16	0.39	Medium
Zhu et al., 2020 [39]	2	4	0.50	4	0.49	Medium

**Table 3:** Frequencies of small association, medium association, and strong association for the age groups, and within each age group for each cognitive domain.

**Age Group: All**	**Small**	**Medium**	**Strong**
Children	4	7	4
Adults	3	1	2
Older adults	1	3	0
**Age Group: Children and Adolescents**	**Small**	**Medium**	**Strong**
Attention/EF	1	1	2
Memory	0	1	0
Global cognition	1	1	0
Academic achievement	2	4	2
**Age Group: Adults**	**Small**	**Medium**	**Strong**
Global cognition	1	0	1
Memory	0	1	1
Attention/EF	2	0	0
**Age Group: Older adults**	**Small**	**Medium**	**Strong**
Global cognition	1	3	0

## Supplementary Materials

The following supporting information can be downloaded at: https://www.mdpi.com/article/10.3390/ijerph191811700/s1, Table S1, Search strategies on scientific database; Table S2, Quality assessment of the available evidence; Table S3, Characteristics of greenspace exposure assessment.
