# Supplementary material for: Long-Term Exposure to Greenspace and Cognitive Function during the Lifespan: A Systematic Review
Source: Int J Environ Res Public Health. 2022 Sep 16;19(18):11700. doi: 10.3390/ijerph191811700 (PMC9517665; doi:10.3390/ijerph191811700)
Supplement: Supplementary file 1 [file ijerph-19-11700-s001.zip › Supplementary_Materials_ijerph-1881606.pdf]

International Journal of
Environmental Research and Public Health

MDPI

Table S1. Search strategy on scientific database.

Scopus	(TITLE-ABS-KEY (greenness) OR TITLE-ABS-KEY (greenspace) OR TITLE-ABS-KEY (greenery) OR TITLE-ABS-KEY (urban forestry) AND TITLE-ABS-KEY (cogn*) OR TITLE-ABS-KEY (memory) OR TITLE-ABS-KEY (attent*) OR TITLE-ABS-KEY (lang*) OR TITLE-ABS-KEY (visuospatial) OR TITLE-ABS-KEY (exec*))
PubMed	((("greenness"[Title/Abstract]) OR ("greenspaces"[Title/Abstract]) OR ("greenery"[Title/Abstract]) OR ("urban forestry"[Title/Abstract])) AND (("cogn*"[Title/Abstract]) OR ("memory"[Title/Abstract]) OR ("attent*"[Title/Abstract]) OR ("lang*"[Title/Abstract]) OR ("visuospatial"[Title/Abstract]) OR ("exec*"[Title/Abstract])))
PsychInfo_TI	TI ((("greenness") OR ("greenspace") OR ("greenery") OR ("urban forestry")) AND (("cogn*") OR ("memory") OR ("attent*") OR ("lang*") OR ("visuospatial") OR ("exec*")))
PsychInfo_AB	AB ((("greenness") OR ("greenspace") OR ("greenery") OR ("urban forestry")) AND (("cogn*") OR ("memory") OR ("attent*") OR ("lang*") OR ("visuospatial") OR ("exec*")))
Psychinfo_KW	KW ((("greenness") OR ("greenspace") OR ("greenery") OR ("urban forestry")) AND (("cogn*") OR ("memory") OR ("attent*") OR ("lang*") OR ("visuospatial") OR ("exec*")))

Table S2. Quality assessment of the available evidence.

Authors, Year	Study Design	Confounding Factors	Statistics	Potential Bias	Multiplicity	Outcome Assessment	GS/BS Assessment	Use of GS/BS	Quality of GS/BS	Effect Size	At least 1y Living in the Area	Score (Absolute Number)	Score (%)	Quality Category
Claesen et al., 2021	1	2	1	1	1	1	1	0	0	1	0	9	64.29	good
Dadvand et al., 2015	2	2	1	1	1	1	1	0	0	1	1	11	78.57	good
Dadvand et al., 2017	2	2	1	1	1	1	1	0	0	1	1	11	78.57	good
Dadvand et al., 2018	1	2	1	1	1	1	1	0	0	1	1	10	71.43	good
Flouri et al., 2019	1	2	1	1	1	1	1	0	0	1	0	9	64.29	good
Hodson et al., 2017	1	1	1	1	1	1	1	0	0	1	0	8	57.14	fair
Jimenez et al., 2022	2	2	1	1	1	1	1	0	0	1	0	10	71.43	good
Kuo et al., 2018	1	1	1	1	1	1	1	0	1	1	0	9	64.29	good
Kuo et al., 2021	1	1	1	1	1	1	1	0	1	1	0	9	64.29	good
Kweon et al., 2017	1	1	1	1	1	1	1	0	1	1	0	9	64.29	good
Leung et al., 2019	1	1	1	1	1	1	1	0	0	1	1	9	64.29	good
Ward et al., 2016	1	1	1	1	1	1	1	1	0	1	1	10	71.43	good
Wu et al., 2014	1	2	1	1	1	1	1	0	0	1	0	9	64.29	good
Sivarajah et al., 2018	1	1	1	1	1	1	1	1	1	1	0	10	71.43	good
Bijnens et al., 2022	1	1	1	1	1	1	1	0	1	1	0	9	64.29	good
Cerin et al., 2021	1	2	1	1	0	1	1	0	0	1	0	8	57.14	fair
Lega et al., 2021	1	2	1	1	1	1	1	0	0	1	0	9	64.29	good
Dzhambov et al., 2019	1	2	1	1	1	1	1	0	0	1	1	10	71.43	good
Zijlema et al., 2017	1	2	1	1	1	1	1	1	0	1	0	10	71.43	good
Hystad et al., 2019	1	1	1	1	0	1	1	0	0	1	1	8	57.14	fair
Crous-Bou et al., 2020	1	2	1	1	0	1	1	0	0	1	0	8	57.14	fair
de Keijzer et al., 2017	2	2	1	1	1	1	1	0	0	1	0	10	71.43	good
Jin et al., 2021	1	2	1	1	1	1	1	0	0	1	0	9	64.29	good
Zhu et al., 2019	2	2	1	1	1	1	1	0	0	1	0	10	71.43	good
Zhu et al., 2020	1	2	1	1	1	1	1	0	0	1	0	9	64.29	good

Table S3. Characteristics of greenspace exposure assessment.

Authors, Year	Level of Greenspace	Greenspace Indicator	Buffer	Physical Access to GS	Use of GS	Time in GS	Quality of GS
Claesen et al., 2021	School surrounding greenness	NDVI	100 m<br>300 m<br>1000 m<br>2000 m	0	0	0	0
Dadvand et al., 2015	Residential surrounding greenness<br>Commuting greenness<br>School greenness	NDVI	250 m (home)<br>50 m (commuting route)<br>50 m (school)	0	0	0	0
Dadvand et al., 2017	Residential surrounding greenness	NDVI<br>VFC	100 m<br>300 m<br>500 m	0	0	0	0
Dadvand et al., 2018	Residential surrounding greenness	NDVI	100 m	0	0	0	0
Flouri et al., 2019	Neighbourhood greenspace	Data from Multiple Environmental Deprivation Index (MEDIx)	No	0	0	0	0
Hodson et al., 2017	School greenness	Average percent canopy cover<br>Average percent impervious surfaces<br>Grass/Shrub cover	No	0	0	0	0
Jimenez et al., 2022	Residential surrounding greenness	NDVI	90 m	0	0	0	0
Leung et al., 2019	Greenness surrounding school	NDVI<br>Green land use	250 m<br>500 m<br>1000 m<br>2000 m	0	0	0	0
Kuo et al., 2018	School and neighbourhood greenness	Tree canopy cover	25 m	0	0	0	0

Kuo et al., 2021	School greenness	Grass/shurb cover<br>NDVI<br>Tree canopy cover	250 m<br>1000 m	0	0	0	1
Kweon et al., 2017	School greenness	Land cover variables	No	0	0	0	1
Ward et al., 2016	Greenspace	Time spent in GS	No	0	0	1	0
Wu et al., 2014	Greenness of school surrounding	NDVI	250 m<br>500 m<br>1000 m<br>2000 m	0	0	0	0
Sivarajah et al., 2018	Vegetation around school	Total land area (m2)<br>Total soft surface (m2)<br>Tree canopy cover (m2)<br>Percentage tree cover	No	0	0	0	1
Bijnens et al., 2022	Residential surrounding greenspace<br>School surrounding greenspace<br>Proximity to accessibile greenspace	Land cover data from the Agency for Geographic Information Flanders	50 m<br>100 m<br>300 m<br>500 m<br>1000 m<br>2000 m	1	0	1	1
Cerin et al., 2021	Parkland in residential buffer	Percentage of parkland in residential buffer	1 km	0	0	0	0
Lega et al., 2021	Residential surrounding greenness	NDVI	400 m	0	0	0	0
Dzhambov et al., 2019	Residential surrounding greenness	NDVI	100 m<br>300 m<br>500 m<br>1000 m	0	0	0	0

Zijlema et al., 2017	Residential surrounding greenness	NDVI<br>Distance to NOE	100 m<br>300 m<br>500 m	1	0	0	0
Hystad et al., 2019	Residential surrounding greenness	NDVI	500 m	0	0	0	0
De Keijzer et al., 2018	Residential surrounding greenness	NDVI<br>EVI	500 m<br>1000 m	0	0	0	0
Crous-Bou et al., 2021	Residential surrounding greenness	NDVI	300 m	0	0	0	0
Jin et al., 2021	Residential surrounding greenness	NDVI	400 m	0	0	0	0
Zhu et al., 2019	Residential surrounding greenness	NDVI	500 m	0	0	0	0
Zhu et al., 2020	Residential surrounding greenness	NDVI	500 m	0	0	0	0

NDVI = Normalized Difference Vegetation Index; VFC = Vegetation Continuous Field; GS = Greenspace; NOE = Natural Outdoor Environment; EVI = Enanched Vegetation Index.
